# Effectiveness of Social Robots in Relieving Children's and Adolescents' Anxiety, Pain, Fear and Stress in Perioperative Nursing: A Systematic Review and Meta‐Analysis

**DOI:** 10.1111/jocn.70411

**Published:** 2026-06-20

**Authors:** Miia Kauppinen, Aku Forsman, Heli Kerimaa, Anna‐Kaija Palomaa, Mervi Hakala, Shingo Ueki, Tarja Pölkki

**Affiliations:** ^1^ Research Unit of Health Sciences and Technology, Faculty of Medicine University of Oulu Oulu Finland; ^2^ The Finnish Centre for Evidence‐Based Healthcare: A Joanna Briggs Institute Centre of Excellence Helsinki Finland; ^3^ Medical Research Center Oulu, Oulu University Hospital University of Oulu Oulu Finland; ^4^ Oulu University Hospital Oulu Finland; ^5^ Department of Health Sciences, Faculty of Medical Sciences Kyushu University Fukuoka Japan; ^6^ JBI Japan Centre for Evidence‐Based Practice: A Joanna Briggs Institute Centre of Cellence Osaka Japan

**Keywords:** adolescents, anxiety, children, fear, meta‐analysis, pain, perioperative nursing, social robots, stress, systematic review

## Abstract

**Aim:**

To assess how social robots impact anxiety, pain, fear and stress in children and adolescents during perioperative nursing.

**Design:**

A systematic review and meta‐analysis of randomised controlled trials and quasi‐experimental studies.

**Methods:**

Following Joanna Briggs Institute guidelines, eligible studies included participants under 21 undergoing surgery or perioperative care, used social robot interventions with comparators, assessed anxiety, pain, fear and stress, and employed randomised or quasi‐experimental designs. Two independent reviewers screened and extracted data.

**Data Sources:**

A systematic search was conducted in April 2024 and updated twice (December 2024, October 2025) across CINAHL, Web of Science, Scopus, PubMed, Cochrane CENTRAL, Medic, PsycArticles, Institute of Electrical and Electronics Engineers and grey literature sources (Google Scholar, Mednar).

**Results:**

Seven studies (663 participants) showed heterogeneity in social robotic interventions. A meta‐analysis of two studies (302 participants) found a significant reduction in children's and adolescents' post‐operative anxiety, whereas a narrative synthesis identified three studies reporting reduced perioperative anxiety. However, the interventions had little or no effect on perioperative pain, fear or stress.

**Conclusion:**

This review suggests that social robotic interventions may reduce perioperative anxiety in children and adolescents, potentially serving as non‐pharmacological distraction tools in paediatric care. Evidence for their effects on pain, fear and stress is limited, highlighting the need for further research to identify effective interventions and clarify their role in perioperative nursing.

**Impact:**

This review highlights social robotic interventions as promising non‐pharmacological tools for reducing perioperative anxiety in children and adolescents.

**Reporting Method:**

Preferred Reporting Items for Systematic Reviews and Meta‐Analyses and Synthesis Without Meta‐Analysis reporting guidelines.

**Patient or Public Contribution:**

No patient or public contribution.

**Trial Registration:**

This review was registered in PROSPERO, the International Prospective Register of Systematic Reviews (https://www.crd.york.ac.uk/PROSPERO/view/CRD42024549796)

## What Does This Paper Contribute to the Wider Global Clinical Community?

- Supports clinical decision‐making in perioperative care by providing evidence‐based insights on using social robots as non‐pharmacological interventions to reduce anxiety in children and adolescents.
- Enhances understanding of which social robotic strategies are effective in paediatric perioperative care, informing the design and implementation of interventions globally.
- Identifies gaps in evidence regarding perioperative pain, fear and stress, highlighting the need for further research on social robotic interventions in nursing.

## 1 Introduction

Every year, millions of children undergo different types of surgical procedures (Rabbitts and Groenewald 2020), which are often stressful experiences and can cause them substantial anxiety, pain and fear (Friedrichsdorf and Goubert 2020). This can occur throughout the preoperative and postoperative continuum, known as the perioperative period (Miller et al. 2014). Perioperative anxiety and pain have been associated with negative outcomes in children, including increased analgesic use, maladaptive behaviour and delayed recovery (Otto et al. 2025; Mathias and Pai 2022). Surgery‐related negative responses, such as anxiety, may be caused, for example, by separation from parents, exposure to unfamiliar clinical environments, pain, and the disorienting effects of anaesthesia (Stanzel and Sierau 2022).

Anxiety is a prevalent issue among children undergoing surgical procedures, affecting up to 75% of paediatric patients (Getahun et al. 2020). It can be characterised by tension, irritability and increased autonomic nervous system activity (Aytekin et al. 2016). Pain is also a common experience in paediatric patients, and its management is still often insufficient (Friedrichsdorf and Goubert 2020), especially during the postoperative stage (Mekonnen et al. 2021). Approximately one‐third of children experience notable pain that persists for up to 1 week following surgery (Fortier and Kain 2015). It is also noteworthy that surgery‐related anxiety and pain in children are often associated, as for example, high levels of perioperative anxiety in children tend to result in greater postoperative pain. Elevated anxiety levels in children enhance the sympathetic nervous system's response to pain stimuli, heightening their perception of pain (Mathias and Pai 2022). Moreover, anxiety and fear of surgery activate the human stress response, which is similar to other stressors such as pain, cold and infection (Zainal Abidin et al. 2021), leading to increased risk of maladaptive behaviour. Thus, a better understanding and ways to improve perioperative management of paediatric patients to promote a calm and positive experience is needed (Ahmed et al. 2011).

Multimodal strategies, both pharmacological and non‐pharmacological, are recommended to manage the negative effects of surgery in children (Sullivan et al. 2024; Friedrichsdorf and Goubert 2020). Especially age‐appropriate distraction is a simple and effective non‐pharmacological method that directs children's attention away from an unpleasant procedure to other stimuli (Bukola and Paula 2017). Distraction methods may, for example, include music, books, movies or games (Birnie et al. 2018), but recently, social robots have also been gaining more interest for such purposes in paediatric healthcare (Triantafyllidis et al. 2023; Trost et al. 2019). They can leverage affective behaviours such as empathy, playfulness and responsiveness and are thus perceived to ease emotional distress and create a more comfortable and engaging healthcare experience (González‐González et al. 2021). This may be particularly useful when addressing the care needs of children and highlights the potential of social robots in paediatric healthcare (Dawe et al. 2019). This is particularly relevant given that children generally respond well to social robots and often prefer their interactive features over standard care (Logan et al. 2019). Interaction with social robots has been found to improve mood, increase engagement and trust, reduce stress in children, and provide greater relaxation and enhanced communication (González‐González et al. 2021).

In medical settings, social robots have been found to mitigate children's stress and anxiety (Smakman et al. 2021) and to reduce pain and fear during medical procedures (Farrier et al. 2020). For example, a randomised controlled study by Beran et al. (2013) found that interaction with a robot during flu vaccinations resulted in significantly less pain and distress in children. Rheel et al. (2022) also demonstrated robot‐led distraction during needle procedures reducing pain intensity in children. However, the results have been conflicting, as some studies have reported only modest or no evidence that social robots alleviate paediatric pain or anxiety, including the randomised controlled trial by Ali et al. (2021) and the study by Logan et al. (2019). For example, Trost et al. (2020) found some indications of social robots' ability to lower children's pain and fear, but the results were not statistically significant. Notably, recent systematic reviews (Pan et al. 2024; Triantafyllidis et al. 2023) indicate that studies on social robots for children's pain and anxiety are largely limited to brief, invasive procedures, such as vaccination or intravenous cannulation. In addition, the research field is relatively new (Mahdi et al. 2022), and studies focusing on the use of social robots in paediatrics are still limited (Triantafyllidis et al. 2023), emphasising the need for further research.

This systematic review provides an innovative perspective on the use of social robots in perioperative nursing, highlighting their potential as non‐pharmacological support to reduce anxiety, pain, fear and stress among children and adolescents. To our knowledge, no previous reviews have examined the scope of such interventions in this context. This is important not only for discovering effective robotic interventions but also for informing evidence‐based perioperative care. Ultimately, these advancements could improve patient experiences, optimise resource utilisation and enhance surgical outcomes.

## 2 The Review

### 2.1 Aims

This systematic review and meta‐analysis aim to evaluate the effectiveness of interventions using social robots to relieve children's anxiety, pain, fear and stress in perioperative nursing, based on evidence from randomised controlled trials and quasi‐experimental studies. The following research questions were addressed:

1. What types of social robot interventions have been implemented to reduce children's anxiety, pain, fear and stress in perioperative nursing?
2. How effective are these interventions in reducing children's anxiety, pain, fear and stress in perioperative nursing?

## 3 Methods

### 3.1 Design

A systematic review and meta‐analysis were conducted according to the Joanna Briggs Institute (JBI) guidelines (Aromataris and Munn 2020) and registered on PROSPERO, the International Prospective Register of Systematic Reviews (CRD42024549796). However, no separate protocol was published prior to the completion of the review. The methods and results of this review were reported in accordance with the Preferred Reporting Items for Systematic Reviews and Meta‐Analyses (PRISMA) guidelines (Page et al. 2021), as well as the Synthesis Without Meta‐analysis (SWiM) guidelines (Campbell et al. 2020).

### 3.2 Search Methods

Searches of eight electronic databases, including CINAHL (EBSCO database), Web of Science, Scopus, PubMed, Cochrane CENTRAL, Medic, PsycArticles and the Institute of Electrical and Electronics Engineers (IEEE) were conducted in April 2024 and updated again in December 2024 and October 2025. The PICOS model (Population, Interventions, Comparators, Outcomes and Study type) was applied in formulating the search strategy and research questions. Prior to the final searches, a scoping search was carried out to identify articles on the topic, the exclusion criteria and the planned keywords. Additional searches were conducted via Google Scholar and the Mednar (Medical Grey Literature Collection) database to identify any relevant research. In addition, the reference lists of all included studies were systematically checked to identify any further relevant studies that may have been missed in the initial search. Searches were limited to English, Finnish and Swedish language publications but no time restrictions were made. The key Medical Subject Headings (MeSH) terms and CINAHL headings were applied in the searches whenever possible. Synonyms were combined using AND/OR operators. The final search terms from each database are presented in Table S1. All the steps of the search process were conducted by two reviewers in close consultation with an experienced librarian in health disciplines.

### 3.3 Inclusion and Exclusion Criteria

The search results were screened and selected in accordance with predefined inclusion and exclusion criteria (Table 1), with two researchers independently assessing them by title, abstract and full text. Any conflicts were resolved through discussion, with the possibility to consult a third reviewer. Any studies that did not meet the inclusion criteria or provide full text were excluded. The study selection process was managed using Covidence systematic review software, which facilitated screening, data extraction and resolution of discrepancies.

**TABLE 1:** Study inclusion criteria according to the PICOS format.

Context	Inclusion criterion
Population (P)	Children or adolescents of ages 0–21 years having some kind of surgical operation or treatment
Intervention (I)	Social robot or socially assistive robot or humanoid robot
Comparators (C)	Control group (standard care or any other intervention)
Outcomes (O)	Children's pain, anxiety, fear and stress
Study design (S)	Randomised Control Trials (RCT) and Quasi‐experimental studies

### 3.4 Search Outcome

A total of 2360 articles were identified from searches conducted at three separate timepoints. Following the first database search (*n* = 1301) and the additional identified literature (*n* = 603), 1280 records remained after duplicate removal and were screened based on title and abstract. Of these, 14 articles were deemed eligible for full‐text assessment, and seven studies were ultimately included in the full review. The initial screening and full‐text assessment were conducted in April 2024. Seven full‐text articles were excluded because they involved the wrong study design (*n* = 4), setting (*n* = 1) or patient population (*n* = 2).

The first update of the literature search was conducted in December 2024, yielding 193 new records, 116 after removal of duplicates. No new full‐text articles met the inclusion criteria. The second update was conducted in October 2025, yielding 263 new records, 114 after removal of duplicates. One full‐text article was assessed but was excluded due to an incorrect setting (*n* = 1). Thus, no additional studies were included in the review from either update. The search and selection process are illustrated in the PRISMA flowchart (Figure 1), which combines the results of all three searches.

**FIGURE 1:**
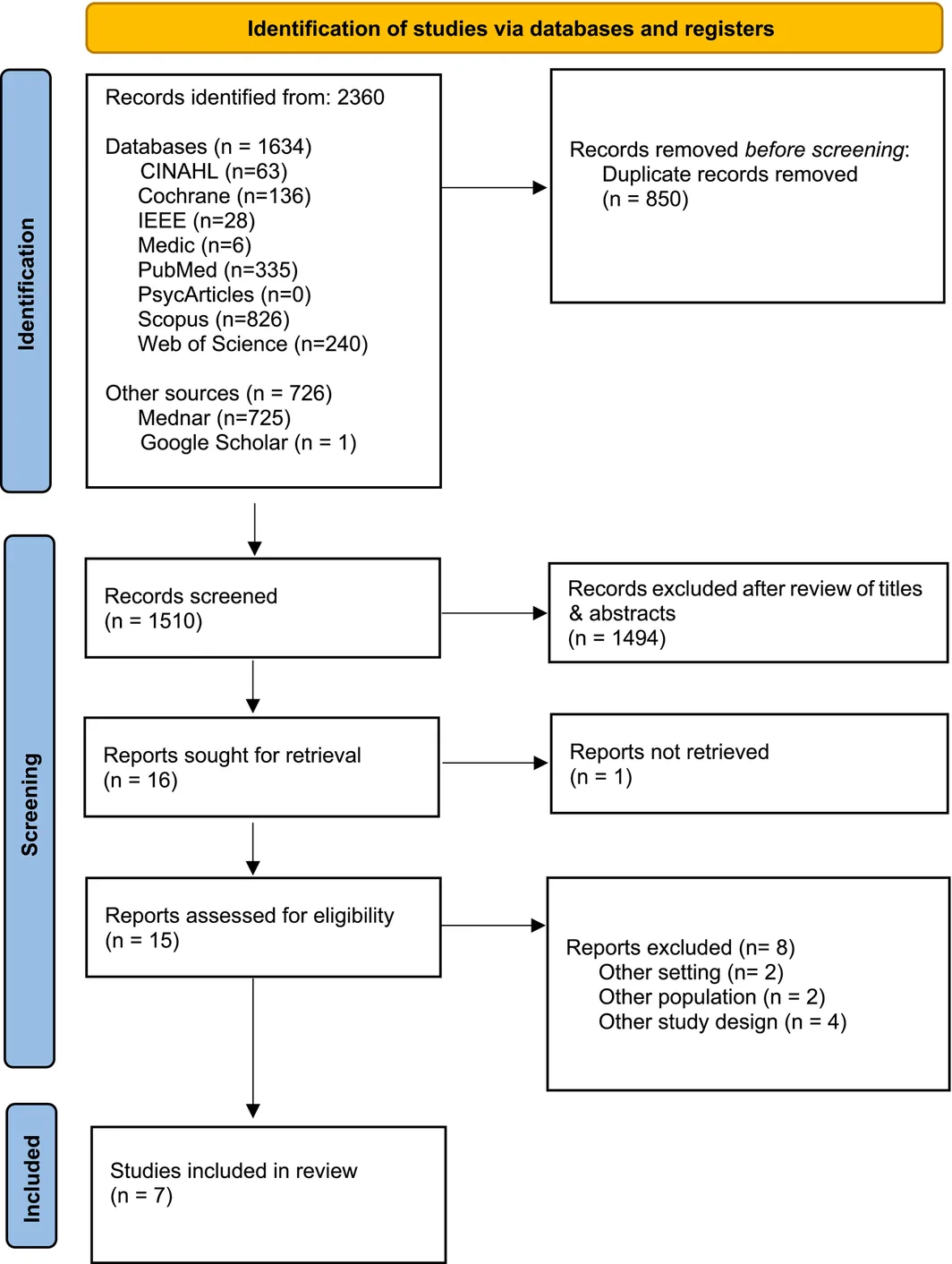
PRISMA flow diagram of the literature search and study selection process. [Colour figure can be viewed at wileyonlinelibrary.com]

### 3.5 Quality Appraisal

The quality of the included studies was appraised by two independent researchers using the standardised critical appraisal instruments from JBI for RCTs (Barker et al. 2023) and the Quality Evaluation Scale (QES) for quasi‐experimental studies (Barker et al. 2024) (Tables 2 and 3). Discrepancies between two reviewers arising from the appraisal were resolved through discussion with the possibility to consult the research team. Regardless of the methodological quality, all the studies were included in the systematic review as this provides a true reflection of the current literature on a specific topic (Barker et al. 2024).

**TABLE 2:** Critical appraisal of the selected studies (randomised controlled trials) according to Joanna Briggs Institute guidelines.

Author	Q1.	Q2.	Q3.	Q4.	Q5.	Q6.	Q7.	Q8.	Q9.	Q10.	Q11.	Q12.	Q13.	Total of ‘Yes’ answers
Kasimoglu et al. (2023)	Y	U	Y	N	N	N	Y	U	Y	Y	Y	Y	Y	8/13
Kasimoglu et al. (2020)	N	U	Y	N	N	U	Y	N	Y	Y	Y	Y	U	6/13
Lee‐Krueger et al. (2021)	Y	Y	Y	N	N	U	Y	U	U	Y	Y	Y	Y	8/13
Topçu et al. (2023)	N	U	Y	N	N	N	Y	Y	Y	Y	Y	Y	Y	8/13

*Note:* Rating scale: yes (Y), no (N), unclear (U), not applicable (NA). Q1. Was true randomisation used for the assignment of participants to treatment groups? Q2. Was allocation to the treatment groups concealed? Q3. Were the treatment groups similar at the baseline? Q4. Were the participants blind to treatment assignment? Q5. Were those delivering the treatment blind to the treatment assignment? Q6. Were the outcomes assessors blind to the treatment assignment? Q7. Were the treatment groups treated identically other than the intervention of interest? Q8. Was follow‐up completed, and if not, were differences between groups in terms of their follow‐up adequately described and analysed? Q9. Were the participants analysed in the groups to which they were randomised? Q10. Were the outcomes measured in the same way for the treatment groups? Q11. Were the outcomes measured in a reliable way? Q12. Was an appropriate statistical analysis used? Q13. Was the trial design appropriate, and any deviations from the standard RCT design (individual randomisation, parallel groups) accounted for in the conduct and analysis of the trial?

**TABLE 3:** Critical appraisal of the selected studies using the Quality Evaluation Scale (QES) for quasi‐experimental studies according to Joanna Briggs Institute guidelines.

Author	QQ1.	QQ2.	QQ3.	QQ4.	QQ5.	QQ6.	QQ7.	QQ8.	QQ9.	Total of ‘Yes’ answers
Beraldo et al. (2019)	Y	N	Y	Y	Y	Y	Y	U	Y	7/9
Franconi et al. (2023)	Y	Y	U	Y	U	Y	Y	U	Y	6/9
Lapedriza et al. (2024)	Y	Y	U	Y	Y	Y	U	N	N	5/9

*Note:* Rating scale: yes (Y), no (N), unclear (U), not applicable (NA). QQ1. Is it clear in the study what is the ‘cause’ and what is the ‘effect’? QQ2. Was there a control group? QQ3. Were participants included in any comparisons similar? QQ4. Were the participants included in any comparisons receiving similar treatment/care, other than the exposure or intervention of interest? QQ5. Were there multiple measurements of the outcome, both pre and post the intervention/exposure? QQ6. Were the outcomes of the participants included in any comparisons measured in the same way? QQ7. Were the outcomes measured in a reliable way? QQ8. Was follow‐up complete and if not, were differences between groups in terms of their follow‐up adequately described and analysed? QQ9. Was an appropriate statistical analysis used?

### 3.6 Data Abstraction

The data were extracted using a modified data extraction template (Centre for Reviews and Dissemination 2009), including key variables such as study characteristics, intervention and control descriptions, outcome measures, measurement instruments, time points, statistical methods and main findings. Two reviewers independently extracted data from all eligible studies. The extraction process covered the PICOS framework and included both study design details and outcome data. The characteristics of the studies included (Table 4) were charted using predefined variables: first author, year of publication, study context and population (including age, size and eligibility criteria), study design, type and content of intervention, outcome measures (e.g., anxiety, pain, fear, stress), time points and data collection methods. The findings were recorded for both interventions and control groups. Moreover, study aims and descriptions of robots used in the interventions were charted separately (Table 5). After independent extraction, the reviewers compared their findings and resolved discrepancies through discussion.

**TABLE 4:** Summary of the included studies.

Authors	Study design	Participants	Setting	Outcomes, measures and time points	Intervention(s)	Data (intervention(s) vs. other(s))	Results
Beraldo et al. (2019)	Quasi‐experimental study, two parallel intervention groups without control Observational preliminary study	Children (*N* = 28) between 3‐ and 19‐ years old undergoing painful procedures, such as arthrocentesis, spinal tap, renal biopsy and bronchoscopy. The Pepper robot group (*N* = 14) and the Sanbot Elf robot group (*N* = 14)	Azienda Ospedaliera of Padua, the Terapia Antalgica e Cure Palliative Pediatriche (Paediatric Pain Therapy and Palliative Care Unit)—Hospice Pediatrico (UOSD), Italy, dates unspecified	Primary outcome: Children's anxiety (a Likert‐type questionnaire) before, during and after procedure Secondary outcome: Children's fear (a Likert‐type questionnaire) before, during and after procedure	In the interventions, either Pepper or Sanbot Elf robots executed pre‐programmed distractions by welcoming the participants, asking questions, and playing guessing games and animations if the child so chose. They also moved to music and expressed emotions. The interactions lasted for few minutes until the sedation took effect. The study did not include a control group	Anxiety during perioperative nursing^b^ Sanbot Elf‐robot: (*χ* ^2^ = 31.764, *p* = 0.003*, *W* = 0.814); Pepper robot: (*χ* ^2^ = 22.543, *p* = 0.047*, *W* = 0.578) Fear during perioperative nursing^b^ Sanbot Elf‐robot: (*χ* ^2^ = 29.559, *p* = 0.005*, *W* = 0.758); Pepper robot: ns	Anxiety: Positive (Sanbot Elf had a stronger overall calming effect) Pain: NM Fear: Positive (but only with Sanbot Elf) Stress: NM
Franconi et al. (2023)	Quasi‐experimental study, two parallel groups Randomisation: According to the day of the week	Children (*N* = 60) between 2‐ and 14‐years old undergoing minor surgery. The intervention (*N* = 30) and control group (*N* = 30)	Azienda Ospedaliero Universitaria delle Marche Hospital, Italy, April 2023 to May 2023	Primary outcome: Children's anxiety (CEMS) pre‐ and intraoperatively	In the intervention group, the robot interacted with the children during premedication and the journey to the surgery wing. It created emotional contact, reassured the participants, and explained the upcoming anaesthesia. During the induction, the robot pretended to blow in a similar mask than the child and played music. In a control group, standard assistance was provided by the personnel	Anxiety during premedication^c^ 6 (5–9) vs. 12 (10–13), *p* = 0.03* Anxiety during anaesthesia 6 (5–8) vs. 10 (8–11), *p* = 0.03* Anxiety across different age groups – Anxiety during premedication < 5‐year‐olds: 8 (5; 16) vs. 13 (7; 20), *p =* 0.052 – Anxiety during premedication 5–7‐year‐olds: 6 (5; 7) vs. 8 (6; 13), *p* = 0.012* – Anxiety during premedication > 7‐year‐olds: 5 (5; 7) vs. 9 (7; 23), *p* = 0.001* – Anxiety during anaesthesia < 5‐year‐olds: 6 (5; 13) vs. 10 (8; 16), *p =* 0.033* – Anxiety during anaesthesia 5–7‐year‐olds: 5 (5; 10) vs. 7 (5; 9), *p* = 0.143 – Anxiety during anaesthesia > 7‐year‐olds: 5 (5; 6) vs. 9 (5; 25), *p* = 0.008*	Anxiety: Positive Pain: NM Fear: NM Stress: NM
Kasimoglu et al. (2023)	RCT, two parallel groups Randomisation: Block design	Children (*N* = 102) between 4‐ and 10‐years old undergoing dental pulpotomy. The intervention (*N* = 50) and control group (*N* = 52)	Istanbul University Faculty of Dentistry, Department of Pedodontics, Turkey, dates unspecified	Primary outcome: Children's anxiety (FIS, pulse) pre‐ and postoperatively Secondary outcome: Children's stress (sAA) pre‐ and postoperatively	In the intervention group, the robot distracted the participants audibly and visually. The control group was treated without the robot distraction. Both groups received alveolar block anaesthesia	Results are presented in meta‐analyses^a^ sAA levels across age groups^d^ – sAA levels preoperatively 4–5‐year‐olds: 233.79 (±69.55) vs. 176.96 (±52.45), *p* = 0.054 – sAA preoperatively 6–10‐year‐olds: 342.27 (±111.79) vs. 310.43 (±144.83), *p* = 0.298 – sAA 1 min postoperative 4–5‐year‐olds: 255.08 (±62.56) vs. 217.9 (±74.13), *p* = 0.275 – sAA 1 min postoperative 6–10‐year‐olds: 360.6 (±121.39) vs. 360.77 (±161.53), *p* = 0.996 – sAA 10 min postoperative 4–5‐year‐olds: 229.01 (±59.91) vs. 209.48 (±69.27), *p* = 0.538 – sAA 10 min postoperative 6–10‐year‐olds: 332.52 (±113.08) vs. 332.71 (±151.92), *p* = 0.995	Anxiety: Positive^a^ Pain: NM Fear: NM Stress: No difference Pulse: Positive^a^
Kasimoglu et al. (2020)	RCT, two parallel groups Randomisation: Not described	Children (*N* = 200) between 4‐ and 10‐years old undergoing dental restoration or pulpotomy. The intervention (*N* = 100) and control group (*N* = 100)	Istanbul University Faculty of Dentistry, Department of Pedodontics, Turkey, dates unspecified	Primary outcome: Children's anxiety (FIS, pulse) pre‐ and postoperatively	In the intervention group, the robot performed some body movements with facial expressions during the treatment. The control group was treated with the basic guidance, distraction and positive reinforcement by the personnel. In both groups, half of the children received local anaesthesia, the rest didn't	Results are presented in meta‐analyses^a^	Anxiety: Positive^a^ Pain: NM Fear: NM Stress: NM Pulse: Positive^a^
Lapedriza et al. (2024)	Quasi‐experimental study, four parallel groups A pilot study	Children (*N* = 86) between 3‐ and 9‐years old undergoing unspecified surgery. A control group called Intervention A (*N* = 31), and intervention groups called Intervention B (*N* = 13), Intervention C (*N* = 30), and Intervention D (*N* = 12)	Care Continuity Unit (CCU) of the Sant Joan de Déu Hospital in Barcelona, Spain, December 2020 to May 2023	Primary outcome: Children's anxiety (STAI, mYPAS) perioperatively	Intervention A: Control group Intervention B: Children waited inside a stationary car Intervention C: Children rode the car ‘interventions’ OFF Intervention D: Children experience the different sensory interventions during the ride	Incomplete data and imbalanced sample size	The quantitative analysis was inconclusive, and thus the results are not presented
Lee‐Krueger et al. (2021)	RCT, two parallel groups Randomisation: Sealed envelopes	Children (*N* = 103) between 4‐ and 12‐years old undergoing elective surgery. The intervention (*N* = 45) and control group (*N* = 58)	A paediatric tertiary healthcare facility in a western Canadian province, Canada, July 2016 to May 2018	Secondary outcome: Children's pain (FPS‐R) intraoperatively Secondary outcome: Children's fear (CFS) pre‐ and intraoperatively	In the intervention group, children received standard preoperative care, including application of Ametop and routine support from healthcare staff, along with a approx. 10‐min session with the MEDi robot. It greeted each child by name and guided deep breathing exercises as a coping strategy for the stressful procedure. The robot also provided interactive encouragement. In the control group, children received only standard care	Pain during IV insertion^d^ – Child: 2.74 (2.96) vs. 2.76 (2.97), *p* = 0.98 – Parent: 2.86 (2.46) vs. 2.70 (2.78), *p* = 0.77 – Research assistant: 2.76 (2.71) vs. 2.67 (2.58), *p* = 0.87 – Anaesthesiologist: 2.55 (2.74) vs. 2.27 (2.69), *p* = 0.62 Pain during propofol bolus – Researcher: 0.93 (2.02) vs. 1.46 (2.60), *p* = 0.29 Fear (procedural phase) before IV^d^ – Child: 1.16 (1.26) vs. 1.13 (1.02), *p* = 0.89 – Parent: 1.41 (1.19) vs. 1.38 (1.05), *p* = 0.88 Fear during IV – Child: 1.39 (1.34) vs. 1.14 (1.13), *p* = 0.33 – Parent: 1.59 (1.06) vs. 1.31 (1.08), *p* = 0.21 – Research assistant: 1.56 (1.08) vs. 1.19 (0.97), *p* = 0.08 – Anaesthesiologist: 1.2 (1.16) vs. 1.12 (1.22), *p* = 0.73 Fear during propofol bolus – Researcher: 0.67 (0.99) vs. 0.65 (0.81), *p* = 0.88	Anxiety: NM Pain: No difference Fear: No difference Stress: NM
Topçu et al. (2023)	RCT, two parallel groups Randomisation: Weekly coin‐flip method	Children (*N* = 84) between 5‐ and 10‐years old undergoing day surgery. The intervention (*N* = 42) and control group (*N* = 42)	A University Hospital, Paediatric Surgery Clinic in northwestern Turkey, June 2020 to April 2022	Primary outcome: Children's state anxiety (CSA) postoperatively	Two hours after surgery, the importance of mobilisation was explained to the children and anxiety levels were measured. In the intervention group, the robot (along with parents and a nurse), accompanied the children's mobilisation efforts by encouraging engagement and diverting attention from postoperative stress. In the control group, only parents and nurses were aiding the children's mobilisation	Anxiety postoperatively^d^ 2.74 (±2.60) vs. 4.50 (±2.96), *p* = 0.005*	Anxiety: Positive Pain: NM Fear: NM Stress: NM

Abbreviations: CEMS, Children's Emotional Manifestation Scale; CFS, Child Fear Scale; CSA, Children's State Anxiety; FIS, Facial Image Scale; FPS‐R, Faces Pain Scale‐Revised; mYPAS, modified Yale Preoperative Anxiety Scale; NM, not measured; pulse, physiological pulse rate (bpm); sAA, salivary alpha amylase; STAI, State‐Trait Anxiety Inventory.

^a^ Results are presented in meta‐analyses.

^b^ Means and standard deviations were not reported with only test statistics and *p*‐values available.

^c^ The adjusted median (95% confidence interval).

^d^ Mean (standard deviation).

* Statistically significant at *p* < 0.05.

**TABLE 5:** Descriptions of the robots used in the interventions.

Study	Type of robot(s)	Description of robot(s)	Aim
Beraldo et al. (2019)	Pepper robot & Sanbot Elf robot	Both robots are hardshelled, humanoid social robots. Pepper (by SoftBank Robotics) is 120 cm tall, whereas Sanbot Elf (by Qihan) measures 90 cm. Both provide distraction by entertaining patients and interacting through voice, gestures, and animated expressions. Sanbot Elf also allows touch interaction. Although Pepper features more complex body movements and vocal tone variation, Sanbot Elf displays a wider range of facial expressions via its screen	To examine the potential of the humanoid social robots as non‐pharmacological techniques for children
Franconi et al. (2023)	NAO robot (named as Estrabot)	A humanoid robot (by Softbank robotics), that speaks in a child's voice and is among the most widely used social robots in human‐robot interaction, due to its affordability and broad functionality. It is hardshelled, 58 cm tall and weighs 5.6 kg, and has features such as text‐to‐speech, speech recognition for 20 languages, object recognition, face recognition, and tracking. The robot can interact with people, dance, move, grab objects and is fully programmable	To determine whether the use of a humanoid robot could reduce preoperative anxiety levels in children
Kasimoglu et al. (2023)	NAO robot	A humanoid, 58 cm tall robot, (by Softbank robotics) able to perform targeted motor tasks (only movement and speech specified). Operated wirelessly via keyboard	To assess the effects of a human‐like robot on the behaviour guidance during children's dental treatment
Kasimoglu et al. (2020)	iRobiQ	A small, hardshelled, humanoid‐type (by Yujin Robot), remotely controlled robot, capable of movement, and different facial expressions through lights. It has a tablet display on its body that allows song, video and animation playback. The robot has can move its head, arms and wheels	To use a humanoid robot as a distraction technique for children to reduce their anxiety and improve their behaviour during dental treatment
Lapedriza et al. (2024)	Robotic ride‐on car	Interactive, hardshelled robotic ride‐on car, customised from a commercial electric toy car by the researchers. Suitable for ages 3–10, remotely controlled. Described as appealing to children and capable of interaction through speech. Plays music, and changes lights. Other features were also designed but were unable to be used in the study	To evaluate the usability of the robotic, ride‐on car to distract paediatric patients before their surgery and reduce their stress levels
Lee‐Krueger et al. (2021)	NAO robot (named as MEDi)	A humanoid robot (by SoftBank Robotics) named MEDi is tablet‐controlled and capable of multimodal interaction through speech and gestures. It uses programmable movement, sound output, and coordinated visual cues to support child engagement	To examine the impact of a humanoid robot on paediatric surgery patients' pain and fear during intravenous (IV) line placement
Topçu et al. (2023)	Silverlit Macrobot	A hardshelled, humanoid‐type robot (by Silverlit Toys), described as appealing, is capable of recording and repeating movements and sounds, responding to hand clapping, following its users, and carrying objects. It can be programmed or operated with a remote control and is suitable for children aged five and older	To evaluate the effect of an interactive robot on children's postoperative anxiety, mobilisation, and parents' satisfaction related to postoperative care

In this review, anxiety was identified as the primary outcome, as it was the most commonly reported and central outcome in evaluating the effects of social robots for children and adolescents in perioperative nursing. The secondary outcomes were defined as pain, fear and stress. In addition to the predefined protocol, one other outcome clearly related to the topic (pulse) was found when examining the studies. Where available, the extracted data included quantitative results such as means, standard deviations, *p*‐values and statistical comparisons.

### 3.7 Data Synthesis

The review employed both a meta‐analysis and a synthesis without a meta‐analysis (SWiM) to synthesise data (Page et al. 2021; Campbell et al. 2020) due to the varying availability of sufficiently homogenous and measurable outcome data. Of the seven studies identified, only two RCTs (Kasimoglu et al. 2023, 2020) were eligible for inclusion in the meta‐analysis, as they reported sufficiently comparable outcome measures, enabling statistical pooling. In both studies, children's and adolescents' pre‐ and postoperative anxiety levels were assessed using the Facial Image Scale (FIS) and outcomes related to physiological stress (pulse in bpm). The Facial Image Scale (FIS) is a tool designed for children to assess their emotional state, such as fear or anxiety, particularly in healthcare settings. It consists of five images depicting facial expressions ranging from happy to fearful, from which the child selects the one that best represents how they feel (Buchanan and Niven 2002).

For the meta‐analysis, data were independently extracted by two reviewers including means, standard deviations (SDs) and sample sizes for both the social robotic intervention and control groups. Mean differences (MDs) with 95% confidence intervals (CIs) were calculated for each outcome using a fixed‐effect model. Heterogeneity was assessed using the *I* ^2^ statistic, with values above 50% considered indicative of substantial heterogeneity. The meta‐analysis was performed using RevMan software version 9.4.0 (Cochrane). Forest plots were generated to visually present the individual and pooled effect estimates, and the results were interpreted in terms of statistical significance and clinical relevance. The Grading of Recommendations Assessment, Development and Evaluation (GRADE) approach was used to assess the certainty of evidence for outcomes included in the meta‐analysis. The results of this assessment are shown in Table S2 created using GRADEpro software (McMaster University, ON, Canada).

The results of five studies (Lapedriza et al. 2024; Franconi et al. 2023; Topçu et al. 2023; Lee‐Krueger et al. 2021; Beraldo et al. 2019) were not suitable for statistical pooling due to differences in their outcome measures, time points or study designs. These studies were synthesised narratively following the SWiM guidelines (Campbell et al. 2020), with the findings being presented descriptively, highlighting the main results and clinical implications of each study. The synthesis was conducted independently by two reviewers. Any discrepancies were resolved through discussion and consensus to minimise subjective interpretation bias and ensure reproducibility. The studies were organised into textual summaries and tables according to outcomes (anxiety measured with other measures, pain, fear and stress), intervention type, and study design, to explore outcome characteristics and variability across the included studies. The findings were synthesised by comparing the reported direction of effect and statistical significance across the included studies.

## 4 Results

### 4.1 Quality and Risk of Bias Appraisal

Overall, the quality of the included studies showed a low to moderate risk of bias, with three out of four RCT studies scoring more than 60% (8/13) of the maximum score on the used critical appraisal tool. Only one RCT (Kasimoglu et al. 2020) received less than 50% (6/13). Notably, true randomisation was applied only in two studies (Kasimoglu et al. 2023; Lee‐Krueger et al. 2021), whereas the other two either used a pseudo‐randomisation method (weekly coin toss) (Topçu et al. 2023) or did not provide enough details (Kasimoglu et al. 2020). Due to the nature of the interventions, that is, children receiving robotic interactions before the outcome measurements were recorded, blinding was not feasible in any of the studies. However, in some studies (Lee‐Krueger et al. 2021) members of the research team (e.g., research assistant, anaesthesiologists) were blinded to the group allocation to minimise bias during data collection. Only one study (Lee‐Krueger et al. 2021) provided enough detail about follow‐up completion. Moreover, in one study (Kasimoglu et al. 2020) the appropriateness of the trial design was deemed unclear, with the study receiving the lowest scores (6/13) in total.

Similarly to the RCTs, the scores for quasi‐experimental studies differed, ranging from 5 to 7 out of the maximum score of 9. One substantial validity limitation was the lack of a control group in one study (Beraldo et al. 2019). In addition, only one of the three studies (Beraldo et al. 2019) provided enough information about the participants, and none of them detailed the follow‐up completion sufficiently. Most notably, one study (Lapedriza et al. 2024) could not complete their quantitative analyses due to incomplete data and an imbalanced sample size, thus the study received the lowest scores (5/9) of the quality appraisal.

### 4.2 Study Characteristics

The detailed characteristics of the included studies are shown in Table 4. This review included four RCTs (Topçu et al. 2023; Kasimoglu et al. 2023, 2020; Lee‐Krueger et al. 2021) and three quasi‐experimental studies (Lapedriza et al. 2024; Franconi et al. 2023; Beraldo et al. 2019). The studies were published from 2019 to 2024 and conducted in Italy (Franconi et al. 2023; Beraldo et al. 2019), Turkey (Topçu et al. 2023; Kasimoglu et al. 2023, 2020), Spain (Lapedriza et al. 2024) and Canada (Lee‐Krueger et al. 2021). A total of 663 paediatric participants were included in the reviewed studies, with sample sizes varying considerably (range: 24–200).

Two of the studies involved dental procedures requiring local anaesthesia (Kasimoglu et al. 2023, 2020), whereas the rest were done in the context of different types of surgeries or painful procedures. Four studies included minor paediatric surgeries (e.g., inguinal hernia, circumcision, tonsillectomy) (Franconi et al. 2023; Topçu et al. 2023; Lee‐Krueger et al. 2021), although Lapedriza et al. (2024) did not specify the type of surgeries. Beraldo et al. (2019) included various painful procedures, such as biopsies, broncho‐ and gastroscopy, spinal taps and venous access.

### 4.3 Characteristics of the Intervention

Different types of social robotic interventions were identified. Six of the studies used some type of humanoid robot (Kasimoglu et al. 2023, 2020; Franconi et al. 2023; Topçu et al. 2023; Lee‐Krueger et al. 2021; Beraldo et al. 2019), whereas one study used an interactive robotic ride‐on car (Lapedriza et al. 2024). It was the only robot designed and customised by the research team, whereas the others were pre‐existing models. All robots included in the reviewed studies were hardshelled designs; none were reported as having soft‐body construction. Detailed features of the robots are shown in Table 5.

Four studies tested their robotic interventions preoperatively; either while the paediatric patients were waiting for their surgery (Lapedriza et al. 2024; Lee‐Krueger et al. 2021) or up until the sedation or anaesthesia was induced (Franconi et al. 2023; Beraldo et al. 2019). Two of the studies tested their robotic interventions during the procedures (dental treatment) (Kasimoglu et al. 2023, 2020) and one conducted the intervention postoperatively (Topçu et al. 2023).

The studies had slightly different settings or contexts in which they were testing the robotic interventions. Two of the studies included elements of preoperative care for minor surgeries or procedures (Franconi et al. 2023; Beraldo et al. 2019), and two studies tested the interventions during paediatric dental treatments (Kasimoglu et al. 2023, 2020). One study was done in a setting of a preoperative transport for paediatric surgeries (Lapedriza et al. 2024), and one tested a robotic intervention in a setting for IV induction for elective surgeries (Lee‐Krueger et al. 2021). One study tested the robotic intervention for post‐operative mobilisation after minor surgery (Topçu et al. 2023). Most of the studies compared the interventions with standard care (Lapedriza et al. 2024; Franconi et al. 2023; Kasimoglu et al. 2023, 2020; Topçu et al. 2023; Lee‐Krueger et al. 2021). However, one study compared two different robotic interventions to each other, without a control (Beraldo et al. 2019).

All the studies provided some form of social robotic distraction for paediatric patients during procedures. Most of them also provided emotional support or encouragement (Franconi et al. 2023; Kasimoglu et al. 2023; Topçu et al. 2023; Lee‐Krueger et al. 2021; Beraldo et al. 2019). The used distraction methods included music or sounds (Lapedriza et al. 2024; Franconi et al. 2023; Topçu et al. 2023; Kasimoglu et al. 2020), discussion and play (Franconi et al. 2023; Beraldo et al. 2019), dancing (Franconi et al. 2023), lights and animation (Lapedriza et al. 2024; Kasimoglu et al. 2020) as well as body movements (Topçu et al. 2023) with facial expressions (Kasimoglu et al. 2023, 2020). In addition, three studies included more interactive, robotic functions with cognitive‐behavioural strategies to help manage children's and adolescents' perioperative negative emotions. In one study, the robot provided basic educational preparation and encouragement for the children and simulated mask anaesthesia during induction (Franconi et al. 2023). In one study, the robot accompanied its users during postoperative mobilisation (Topçu et al. 2023), whereas in another one, the robot guided deep breathing exercises and provided interactive encouragement as a coping strategy for children during a stressful procedure (Lee‐Krueger et al. 2021).

### 4.4 Outcome Measures

Six out of seven studies measured children's and adolescents' perioperative anxiety using different reliable anxiety scales: the Children's State Anxiety (CSA) (Topçu et al. 2023), the Facial Image Scale (FIS) (Kasimoglu et al. 2023, 2020) and the State‐Trait Anxiety Inventory (STAI) (Lapedriza et al. 2024) measure children's anxiety and provide patient‐related self‐reported outcomes. The Children's Emotional Manifestation Scale (CEMS) (Franconi et al. 2023) and the modified Yale Preoperative Anxiety Scale (mYPAS) (Lapedriza et al. 2024) are observational anxiety scales rated by staff or family members. One study used a self‐reported, 5‐point Likert‐type scale questionnaire for anxiety created by the authors (Beraldo et al. 2019).

Children's perioperative pain was measured in one study (Lee‐Krueger et al. 2021) using a modified version of the Faces Pain Scale‐Revised (FPS‐R). It includes both paediatric patient self‐reported outcomes and observational pain scales rated by their parents, research assistants and anaesthesiologists. Fear was measured in two studies. In Lee‐Krueger et al. (2021), the Child Fear Scale (CFS) was used in the same manner as the pain scale. Beraldo et al. (2019) measured fear using the same questionnaire as they did for anxiety.

Additionally, in two of the studies (Kasimoglu et al. 2023, 2020), anxiety or stress was assessed based on changes in physiological indicators by measuring children's pulse rates (bpm). However, neither study explicitly stated what outcome the pulse rate measured, only implying anxiety or stress. In this context, the pulse is considered to be a measure of anxiety, as Kasimoglu et al. (2020) report their pulse‐related findings followed by a discussion on anxiety. One study also measured children's salivary alpha‐amylase (sAA) levels to evaluate anxiety or stress, again using the terms interchangeably. Here, the sAA levels are considered a measure of stress because the authors cite several studies stating the same (Kasimoglu et al. 2023).

### 4.5 Effectiveness of the Interventions

#### 4.5.1 Meta‐Analysis

Meta‐analyses were conducted based on two studies by Kasimoglu et al. (2023, 2020) that applied comparable FIS measures and pulse ratings to evaluate the effectiveness of social robotic interventions on children's anxiety pre‐ and postoperatively. In Kasimoglu et al. (2023), outcomes were reported for two age groups (4–5 years and 6–10 years), which were treated as independent datasets. Consequently, three datasets were included in each meta‐analysis. Separate meta‐analyses were then conducted for each outcome (anxiety and pulse) at each time point (pre‐ and postoperative), resulting in four meta‐analyses with forest plots. The remaining five studies were excluded from the meta‐analyses due to significant discrepancies in outcome measures and assessment timing, which precluded direct comparison and pooling of effect sizes.

The meta‐analysis of preoperative anxiety showed a modest but non‐significant difference between groups, with the intervention group experiencing lower anxiety than the control group (*z* test = 1.78, *p* = 0.08; MD = −0.25, 95% CI: −0.53, 0.03) (Figure 2a). In contrast, the meta‐analysis of postoperative anxiety showed a significant difference between groups, with lower anxiety in the intervention group than in the control group (*z* test = 3.61, *p* = 0.0003; MD = −0.51, 95% CI: −0.78, −0.23) (Figure 2b). Both meta‐analyses demonstrated very little heterogeneity (*I* ^2^ = 0%, Figure 2). The forest plots of the effect estimates of the original studies and the overall effect sizes are presented in Figure 2.

**FIGURE 2:**
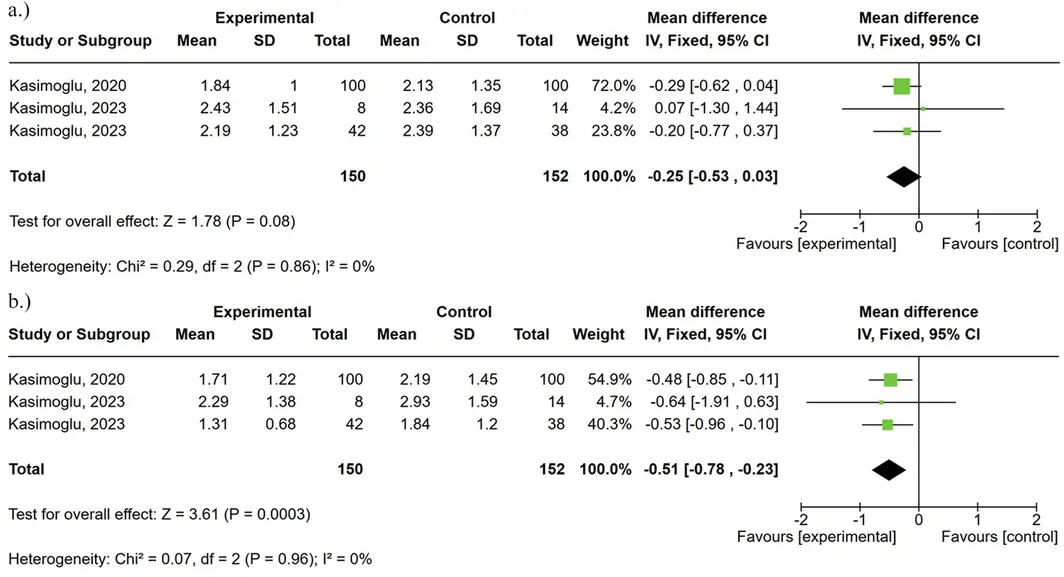
(a) A forest plot of the studies and study subgroups included in the meta‐analysis concerning children's anxiety preoperatively. The figure shows the mean differences between the control and intervention groups across presented interventions and datasets. (b) A forest plot of the studies and study subgroups included in the meta‐analysis concerning children's anxiety postoperatively. In Kasimoglu et al. (2023), outcomes were reported for two age groups (4–5 years and 6–10 years). [Colour figure can be viewed at wileyonlinelibrary.com]

Two additional meta‐analyses were conducted on children's pre‐ and postoperative pulse ratings (Figure 3). Similar to the anxiety findings, preoperative pulse results were not significant (*z* test = 1.06, *p* = 0.29; MD = −2.01, 95% CI: −5.73, 1.71) (Figure 3a). The postoperative values differed significantly between the groups, with lower pulse rates in the intervention group than in the control group (*z* test = 4.90, *p* < 0.00001; MD = −8.41, 95% CI: −11.77, −5.05) (Figure 3b). Heterogeneity was high in the preoperative analysis (*χ* ^2^ = 4.10, *I* ^2^ = 51%, *p* = 0.13), but low in the postoperative analysis (*χ* ^2^ = 2.40, *I* ^2^ = 17%, *p* = 0.30).

**FIGURE 3:**
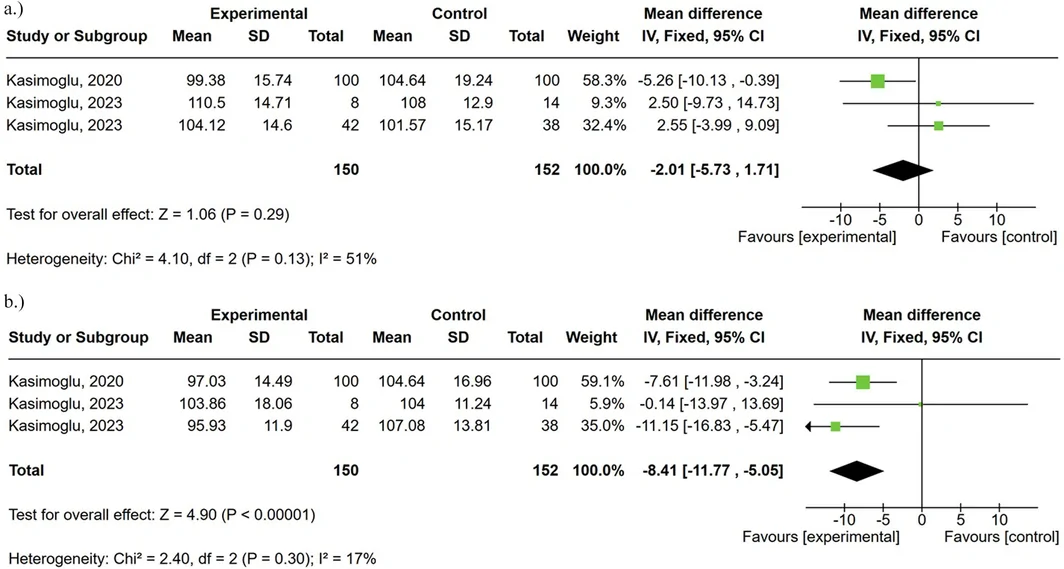
(a) A forest plot of the studies and study subgroups included in the meta‐analysis concerning children's pulse preoperatively. (b) A forest plot of the studies and study subgroups included in the meta‐analysis concerning children's pulse postoperatively. In Kasimoglu et al. (2023), outcomes were reported for two age groups (4–5 years and 6–10 years). [Colour figure can be viewed at wileyonlinelibrary.com]

The results of the GRADE assessment are presented in Table S2. The certainty of evidence for the effects of social robotic interventions on postoperative anxiety and postoperative pulse rate was rated as moderate, whereas the certainty of evidence for their effects on preoperative anxiety was rated as low. Conversely, the certainty of evidence for the effect of social robotic interventions on preoperative pulse rate was rated as very low, reflecting substantial uncertainty due to methodological limitations, imprecision and heterogeneity.

#### 4.5.2 Synthesis Without Meta‐Analysis

Four anxiety‐related studies were not included in the meta‐analysis because of the differences in the anxiety measurements or time points in which the measurements were taken (Lapedriza et al. 2024; Franconi et al. 2023; Topçu et al. 2023; Beraldo et al. 2019). Three of these studies reported statistically significant effects on children's and adolescents' anxiety (Table 4). A study comparing two robotic interventions (Beraldo et al. 2019) found that both robots were associated with statistically significant reductions in children's anxiety across three time points (before, during and after the procedure), with reported *p*‐values of 0.003 for ‘Sanbot Elf’ and 0.047 for ‘Pepper’ (Beraldo et al. 2019). Another study showed that the robotic intervention significantly reduced children's anxiety compared with the control group, both during preoperative (*p* = 0.03) and intraoperative nursing (*p* = 0.03). The results remained similar when comparing different age groups (Table 4) (Franconi et al. 2023). The third included study (Topçu et al. 2023) also reported a significant reduction in children's and adolescents' anxiety in the robotic intervention group compared with the control group (*p* = 0.005), but only during postoperative nursing.

The secondary outcomes considered in the current systematic review were pain, fear and stress experienced by children and adolescents. Out of the seven included studies, only one examined the effectiveness of a robotic intervention on pain relief in children and adolescents in perioperative nursing (Lee‐Krueger et al. 2021). The study did not find any statistically significant differences between the robotic intervention and standard care in relation to children's and adolescents' pain intraoperatively. This was the case for all the raters, that is the children, their parents, the research assistants and researchers, and the anaesthesiologists (Table 4). However, two studies assessed children's fear in relation to robotic interventions intraoperatively, revealing mixed results. Consistent with the pain findings, the first study (Lee‐Krueger et al. 2021) reported that a robotic intervention did not alleviate children's fear before or during anaesthesia induction (Table 4). On the other hand, the second study (Beraldo et al. 2019) reported that fear significantly decreased over time, but only for the ‘Sanbot Elf’ robot group (*p* = 0.005), whereas the change was not statistically significant for the ‘Pepper’ group. This suggests that the ‘Sanbot Elf’ robot may have been more effective in alleviating perioperative fear. Moreover, only one study (Kasimoglu et al. 2023) measured children's perioperative stress, using salivary alpha‐amylase levels in two distinct age groups, and found no detectable differences between the intervention and control groups.

## 5 Discussion

This review examined social robotic interventions aimed at reducing children's anxiety, pain, fear and stress in perioperative nursing. The interventions varied from interactive humanoid robots providing emotional support to task‐oriented robots guiding children through medical procedures. Evidence showed that these robots were particularly effective in reducing postoperative anxiety through engaging interactions. However, findings related to pain, fear and stress were more limited. Overall, social robots appear to be promising tools in paediatric perioperative care, but further research is needed to identify the most effective interventions for various emotional and physiological outcomes.

Although the available data are still scant, social robots appear to offer valuable distraction techniques, an approach that has increasingly been acknowledged as beneficial in paediatric healthcare settings (Pan et al. 2024; Dawe et al. 2019). The interventions included four RCTs and three quasi‐experimental studies featuring interactive humanoid robots, or in one case, a ride‐on robotic car (Lapedriza et al. 2024). Across these studies, the robots interacted with children in various ways. Some interventions provided mere distraction, such as playing, singing or dancing, whereas others featured more immersive engagement with cognitive‐behavioural strategies, such as active engagement, education and teaching of coping strategies during stressful medical procedures. These deeper level interactions appear to be an important differentiator: prior research suggests that passive distraction may be less engaging and less effective than emotionally immersive interventions (Ferraz‐Torres et al. 2022; Trost et al. 2020). However, in this review, both distraction and cognitive‐behavioural strategies showed positive effects in some of the studies, although the findings were not consistent across all.

Both the meta‐analysis and narrative synthesis indicate that social robotic interventions may reduce perioperative anxiety in children. The meta‐analyses pointed to reduced anxiety and pulse rates, with statistically significant effects observed in postoperative nursing. Preoperative anxiety also showed marginally significant improvements, suggesting a positive role for social robots before procedures. Similarly, the narrative synthesis showed three of the four studies with positive effects. These findings are consistent with recent systematic reviews, which demonstrate that social robots can effectively alleviate children's anxiety in clinical settings (Or et al. 2025; Wu et al. 2025; Pan et al. 2024). Furthermore, original empirical studies provide converging evidence across a range of contexts, including minor medical procedures (Neerincx et al. 2025; Chang et al. 2023; Smakman et al. 2021; Rossi et al. 2020), paediatric cancer care (Alemi et al. 2016) and autism interventions (Xie and Park 2024). Although Logan et al. (2019) did not note reduced anxiety, they reported increased positive affect and decreased emotional negativity related to robot use.

The observed anxiety reduction probably stems from several interacting mechanisms: interactive distraction, emotional engagement, predictability and empathy‐like responses provided by the social robots that foster a sense of security. Prior reviews highlight children's reduced anxiety in clinical settings due to emotionally supportive and affectionate robot behaviours (Dawe et al. 2019) and emphasise the importance of predictable and empathetic interactions in fostering emotional safety (Lindsay et al. 2024). However, more mechanistic research is needed to decode precisely how different social robot designs and interaction styles affect psychological outcomes.

The evidence on secondary outcomes is still inconclusive. Pain was assessed in only one study (Lee‐Krueger et al. 2021), which found no differences between the intervention and control groups. Similarly mixed results emerged for fear: Lee‐Krueger et al. (2021) reported no significant effect, whereas Beraldo et al. (2019) found a meaningful reduction in preoperative fear. Consistent with these findings, a review by Pan et al. (2024) also did not observe consistent robot‐related improvements regarding pain or fear. Selective findings elsewhere (Smakman et al. 2021; Farrier et al. 2020; Rossi et al. 2020) suggest that under certain conditions, possibly with specific robot‐intervention designs, children may benefit. Yet, given the limited number of studies, these results should be interpreted cautiously. Low baseline levels, suboptimal timing of intervention or confounding factors like topical anaesthesia (Lee‐Krueger et al. 2021) may have masked potential effects. Moreover, the children's age could have affected the results, as previous research (Smakman et al. 2021) indicates that mid‐primary school children (ages 6–9) may experience the greatest benefit from social robot use in terms of anxiety and pain reduction. In this review, neither study concentrating on the secondary outcomes examined the effects of robots in relation to the children's ages.

The last secondary outcome, stress, was explored only in one of the included studies (Kasimoglu et al. 2023) as a separate outcome with no detectable effects. This is in contrast with previous research, which has shown social robots alleviating children's distress under minor medical procedures (e.g., Pan et al. 2024). However, saliva samples with sAA, as the means of measuring stress in Kasimoglu et al. (2023), may not have been sufficient to detect positive effects. Notably, stress was also mentioned in some of the other included studies (Lapedriza et al. 2024; Kasimoglu et al. 2020), in the context of anxiety, using the terminology somewhat interchangeably. This underscores a need for clearer definitions and standardised measures when examining perioperative emotional states. Future research should distinguish between anxiety, stress and distress more explicitly.

In evaluating postoperative anxiety, it is key to recognise that Topçu et al. (2023) examined anxiety toward mobilisation rather than surgery itself. The study was included, as mobilisation is a core component of postoperative care (Tazreean et al. 2021), but the results should be interpreted with caution. Moreover, Lapedriza et al. (2024) studied preoperative anxiety related to a robotic car intervention but could not provide statistical results due to incomplete data. However, their study was included in this review, as McKenzie et al. (2019) recommend including studies regardless of whether they report outcome data to avoid bias from selective reporting.

To our knowledge, this is the first systematic review investigating the effects of social robotic interventions on children's and adolescents' anxiety, pain, fear and stress in perioperative nursing. These findings can be interpreted within existing paediatric care guidelines, which emphasise child‐centred, developmentally appropriate, and non‐pharmacological approaches to reducing perioperative anxiety (World Health Organisation 2018; National Institute for Health and Care Excellence 2014). In this context, social robots may offer a novel, digitally supported means of delivering these principles. At the national and European levels, healthcare policies also encourage the adoption of innovative technologies to improve quality, safety and patient experience (Ministry of Social Affairs and Health 2023; European Commission 2021). Despite these preliminary but promising findings, however, the current body of evidence remains limited in scope and depth. The heterogeneity of methodologies, small sample sizes, variability in interventions and age groups, reliance on self‐reported outcomes, and lack of long‐term follow‐up data all warrant cautious interpretation of the results.

### 5.1 Strengths and Limitations

Reporting of the methodology and results followed the PRISMA 2020 Checklist (Appendix S1) and the PRISMA 2020 abstract guidelines (Page et al. 2021). The search strategy was created with the help of an information specialist, and the strategy was tested before conducting the final search. The systematic selection process of original studies was carried out in three phases (titles, abstracts and full texts) after duplicate removal, to ensure impartiality, quality and transparency (Liberati et al. 2009). To further improve the transparency of the review, the Joanna Briggs Institute's (JBI) critical appraisal tools for RCTs (Barker et al. 2023) and quasi‐experimental studies (Barker et al. 2024) were used in this review. The inclusion and critical appraisal of the literature, as well as data extraction and synthesis, were conducted by two reviewers independently, with the possibility to consult a third reviewer. The outcome data were analysed using a meta‐analysis (Higgins et al. 2019), and when not feasible, the SWiM approach was applied to ensure a systematic and transparent synthesis of the findings (Campbell et al. 2020). The meta‐analysis was also reviewed by a statistician.

This review has certain limitations that should be considered when interpreting its findings. The included studies exhibited considerable heterogeneity in methodology, interventions, outcome variables and timing of measurements (pre‐, intra‐ and post‐operative). Heterogeneity was also evident in the characteristics of both the robots, such as differing models and types, and the paediatric populations, with variation in age and types of surgeries. This variability, together with generally small sample sizes, constrains the generalisability of the findings. In addition, the limited number of studies, despite extensive database and manual searches, resulted in only two studies being suitable for meta‐analysis. This was also reflected in the GRADE assessment, in which the certainty of evidence varied across outcomes and was limited by methodological concerns, imprecision and heterogeneity. Furthermore, as trial registries were not searched, additional relevant studies may have been missed.

None of the studies were able to blind participants due to the nature of the interventions and the reliance on self‐reported outcomes, which may have influenced participants' reported responses. Only one study (Lee‐Krueger et al. 2021) employed additional blinding procedures for research personnel. The use of self‐reports may increase the risk of bias (Rosenman et al. 2011), although they remain essential for capturing children's subjective experiences, such as pain and anxiety (Bukola and Paula 2017). It is also noteworthy that one included study (Beraldo et al. 2019) did not report numerical means or standard deviations, providing only test statistics and *p*‐values.

The use of pulse as an outcome measure also presents a limitation worth noting, as its relevance to anxiety is not clearly justified in the original studies (Kasimoglu et al. 2023, 2020), and physiological indicators cannot be reliably attributed to specific emotions. Although changes in pulse may reflect procedural effects, the findings derived from this measure, including those in the meta‐analysis, should be interpreted with caution. In addition, none of the studies assessed the longer‐term effects of social robot interventions beyond the day of surgery, limiting conclusions about sustained outcomes. The observed benefits may also be influenced by factors such as the novelty of interacting with social robots or the increased attention provided by researchers during the intervention, thereby limiting the extent to which these effects can be expected to persist over time.

### 5.2 Implications for Practice and Further Research

As a rapidly growing and evolving field (Mahdi et al. 2022), social robotics has the potential to address several key healthcare challenges in the future, particularly in areas where human resources are limited or where supportive interaction could enhance outcomes, such as in paediatric healthcare. The findings of this review show that social robotic interventions in surgical contexts vary, and the overall effectiveness of the interventions for paediatric patients was mixed. However, the findings suggest that social robotic interventions have the potential to reduce perioperative anxiety in children and adolescents. In this context, social robots may be considered as supportive, non‐pharmacological tools within paediatric perioperative care, particularly during postoperative recovery.

Given the heterogeneity of the interventions, there is a clear need for greater consistency in how social robots are implemented. Especially, the need for high quality, randomised controlled trials is clear to minimise confounding and allow for better comparisons of interventions. Developing structured approaches for robot use (e.g., considering duration, timing and type of interaction) could help make interventions more comparable and reproducible. For policymakers and healthcare administrators, the findings suggest that supporting pilot programmes within paediatric surgical units may be valuable, alongside careful evaluation of clinical effectiveness, cost‐effectiveness and patient‐reported outcomes. Establishing guidance around data handling, safety and the potential role of robots within care teams could also support safe and practical implementation. A multidisciplinary approach between robotics and healthcare professionals would help ensure that interventions are both clinically relevant and technically feasible. In practice, social robots could be designed to allow personalisation based on patient age, clinical context and individual preferences, with implementation ideally supported by adequate infrastructure, staff training and integration into existing clinical workflows (Ragno et al. 2023).

Although social robotic interventions show promise in reducing perioperative anxiety, the findings of this review indicate that research on their use in paediatric healthcare, particularly in perioperative nursing, remains limited, pointing to the need for further investigation. Future studies should explore the long‐term effects of social robotic interventions on children, both in general and in surgical contexts, as this remains an underexplored area (Triantafyllidis et al. 2023). In addition, all robots included in this review were hard‐shelled designs, with no studies examining soft‐bodied alternatives. Future research could therefore investigate whether soft‐bodied designs, which may offer greater flexibility and more natural interaction, influence clinical outcomes differently.

## 6 Conclusions

The current systematic review shows that only a limited number of studies have investigated the effects of social robots on children's and adolescents' anxiety, pain, fear and stress in perioperative nursing. This suggests that the use of social robots in relation to surgery is still relatively rare. Despite limited evidence, several social robotic interventions were identified for these purposes. The results of this review also indicate that social robotic interventions can be an effective non‐pharmacological distraction tool for reducing children's perioperative anxiety. Given that surgery can cause significant anxiety in children (Getahun et al. 2020), these findings highlight the potential of the use of social robots in paediatric surgery contexts. However, this review shows little evidence of the effectiveness of reducing children's perioperative pain or stress using similar interventions. There is a clear need for more rigorous research to determine the effectiveness of social robots used for these purposes, as well as to investigate the possibility of integrating social robots in paediatric surgery settings.

## Author Contributions

Miia Kauppinen, Aku Forsman, Heli Kerimaa and Tarja Pölkki were responsible for the conception of the research topic and design. Miia Kauppinen and Aku Forsman performed the data collection and were responsible for the quality assessment of the included studies. Shingo Ueki performed the meta‐analysis. Miia Kauppinen and Aku Forsman performed the data synthesis and were responsible for drafting the manuscript. Heli Kerimaa, Mervi Hakala, Anna‐Kaija Palomaa, Shingo Ueki and Tarja Pölkki made critical revisions to the paper for important intellectual content. Heli Kerimaa and Tarja Pölkki supervised the study.

## Funding

The authors have nothing to report.

## Conflicts of Interest

The authors declare no conflicts of interest.

## Supporting information


**Table S1:** Search terms used across databases and other sources.

**Table S2:** Summary of findings.

**Appendix S1:** PRISMA 2020 Checklist.

## Data Availability

The data that support the findings of this study are available on request from the corresponding author. The data are not publicly available due to privacy or ethical restrictions.
