# Supplementary material for: Effectiveness of Social Robots in Relieving Children's and Adolescents' Anxiety, Pain, Fear and Stress in Perioperative Nursing: A Systematic Review and Meta‐Analysis
Source: J Clin Nurs. 2026 Jun 20;35(11):4725–42. doi: 10.1111/jocn.70411 (PMC13629257; doi:10.1111/jocn.70411)
Supplement: Supplementary file 2 [file JOCN-35-4725-s002.docx]

| Social robotic intervention compared with usual care for reducing perioperative anxiety and pulse rate in children | | | | |
| --- | --- | --- | --- | --- |
| **Population:** Children or adolescents of ages 0 – 21 years having some kind of surgical operation or treatment  **Intervention:** Social robot or socially assistive robot or humanoid robot  **Comparator:** Control group (standard care or any other intervention)  **Outcome:** Pre- and postoperative anxiety and pulse rate included in the meta-analysis | | | | |
| **Outcomes** | **Effect estimate**  **(MD, 95 % CI)** | **№ of participants (studies)** | **Certainty (GRADE)** | **Comments** |
| **Preoperative anxiety**  **(measured with FIS)** | **MD −0.25**  (95% CI −0.53 to 0.03) | 302  (2 RCTs; 3 datasets) | **⊕⊕◯◯**  **LOW**^a,b^ | Certainty was rated low because both included studies had methodological concerns, and the pooled estimate was imprecise. |
| **Postoperative anxiety**  **(measured with FIS)** | **MD −0.51**  (95% CI −0.78 to −0.23) | 302  (2 RCTs; 3 datasets) | **⊕⊕⊕◯**  **MODERATE**^a^ | Certainty was rated moderate because both included studies had methodological concerns, which lowers confidence in the estimate. |
| **Preoperative pulse rate**  **(beats/min)** | **MD −2.01**  (95% CI −5.73 to 1.71) | 302  (2 RCTs; 3 datasets) | **⊕◯◯◯**  **VERY LOW**^a,b,c^ | Certainty was rated very low because both included studies had methodological concerns, heterogeneity was substantial, and the pooled estimate was imprecise. |
| **Postoperative pulse rate**  **(beats/min)** | **MD −8.41**  (95% CI −11.77 to −5.05) | 302  (2 RCTs; 3 datasets) | **⊕⊕⊕◯**  **MODERATE**^a^ | Certainty was rated moderate because both included studies had methodological concerns, which lowers confidence in the estimate. |

**Explanations**

**ᵃ.** Downgraded one level for risk of bias. Both included studies were judged as having 'some concerns' on the JBI Critical Appraisal Checklist for RCTs. In Kasimoglu 2020, the method of randomisation was not adequately reported, and both allocation concealment and blinding were unclear. In Kasimoglu 2023, allocation concealment was unclear and blinding of participants/personnel was not performed.

**ᵇ.** Downgraded one level for imprecision. The 95% confidence interval crosses the line of no effect, making the result compatible with both a clinically meaningful benefit and no effect. The pooled estimate is therefore underpowered to confirm or exclude a meaningful difference.

**ᶜ.** Downgraded one level for inconsistency. Between-study heterogeneity was substantial (I² = 51%), indicating that the magnitude—and to a lesser extent the direction—of effect varied across datasets.

**GRADE Working Group certainty of evidence**

**High:** We are very confident that the true effect lies close to that of the estimate of the effect.

**Moderate:** We are moderately confident in the effect estimate; the true effect is likely close to the estimate, but there is a possibility that it is substantially different.

**Low:** Our confidence in the effect estimate is limited; the true effect may be substantially different from the estimate.

**Very low:** We have very little confidence in the effect estimate; the true effect is likely to be substantially different from the estimate.

**Abbreviations**

CI, confidence interval; FIS, Facial Image Scale; GRADE, Grading of Recommendations Assessment, Development and Evaluation; I², I-squared (heterogeneity statistic); JBI, Joanna Briggs Institute; MD, mean difference; RCT, randomised controlled trial.

Table S2. Summary of Findings.
