# Supplementary material for: Effectiveness of Social Robots in Relieving Children's and Adolescents' Anxiety, Pain, Fear and Stress in Perioperative Nursing: A Systematic Review and Meta‐Analysis
Source: J Clin Nurs. 2026 Jun 20;35(11):4725–42. doi: 10.1111/jocn.70411 (PMC13629257; doi:10.1111/jocn.70411)
Supplement: Supplementary file 3 [file JOCN-35-4725-s003.docx]

| **Database** | **Search terms** |
| --- | --- |
| CENTRAL | (([mh "Robotics"]) OR (robotic*):ti,ab,kw) AND (([mh "Child"]) OR ([mh "Adolescent"]) OR ([mh "Pediatric Nursing"]) OR (child* OR pediatric* OR paediatric* OR adolescent*):ti,ab,kw) AND (([mh "Pain"]) OR ([mh "Pain Management"]) OR ([mh "Stress, Psychological"]) OR ([mh "Anxiety"]) OR ([mh "Fear"]) OR (pain* OR fear* OR stress* OR anxiety*):ti,ab,kw) AND (([mh "Perioperative Care"]) OR ([mh "Perioperative Period"]) OR ([mh "Surgical Procedures, Operative"]) OR ([mh "Ambulatory Surgical Procedures"]) OR (perioperative OR intraoperative OR preoperative OR postoperative OR "operative time" OR "anesthesia recovery period" OR surg* OR "operative room nursing" OR "postanesthesia nursing"):ti,ab,kw) |
| CINAHL | ((MH "Robotics") OR robot*) AND ((MH "Child+") OR (MH "Adolescence+") OR (MH "Pediatric Nursing+") OR (MH "Pediatric Care+") OR (child* OR pediatric* OR adolescen*)) AND ((MH "Pain+") OR (MH "Stress+") OR (MH "Fear+") OR (MH "Anxiety+") OR (MH "Pain Management") OR (pain* OR fear* OR stress* OR anxiety)) AND (((MH "Perioperative Nursing+") OR (MH "Perioperative Care+") OR (MH "Surgery, Operative+")) OR (perioperative OR intraoperative OR preoperative OR postoperative OR “operative time” OR “anesthesia recovery period” OR surg* OR “operative room nursing” OR “postanesthesia nursing”)) |
| IEEE Xplore | robot* AND child* OR pediatric* OR paediatric* OR adolescen* AND pain* OR fear* OR stress* OR anxiety AND perioperative OR intraoperative OR preoperative OR postoperative OR “operative time” OR “anesthesia recovery period” OR surg* OR “operative room nursing” OR “postanesthesia nursing” |
| Medic | robot* AND Pain Stress* Anxiety Fear kipu* kivu* pelko* pelo* ahdistu* |
| Pubmed | ((("Robotics"[Mesh:NoExp]) OR (robot*[Title/Abstract]))) AND (((("Child"[Mesh]) OR "Adolescent"[Mesh]) OR "Pediatric Nursing"[Mesh]) OR (child*[Title/Abstract] OR pediatric*[Title/Abstract] OR adolescen*[Title/Abstract]))) AND (((((("Pain"[Mesh]) OR "Pain Management"[Mesh]) OR "Stress, Psychological"[Mesh]) OR "Anxiety"[Mesh]) OR "Fear"[Mesh]) OR (pain*[Title/Abstract] OR fear*[Title/Abstract] OR stress*[Title/Abstract] OR anxiety[Title/Abstract]))) AND ((((("Perioperative Care"[Mesh]) OR "Perioperative Period"[Mesh]) OR "Surgical Procedures, Operative"[Mesh]) OR "Ambulatory Surgical Procedures"[Mesh]) OR (perioperative[Title/Abstract] OR intraoperative[Title/Abstract] OR preoperative[Title/Abstract] OR postoperative[Title/Abstract] OR "operative time"[Title/Abstract] OR "anesthesia recovery period"[Title/Abstract] OR surg*[Title/Abstract] OR "operative room nursing"[Title/Abstract] OR "postanesthesia nursing"[Title/Abstract])) |
| Scopus | robot* AND child* OR pediatric* OR adolescen* AND pain* OR fear* OR stress* OR anxiety AND perioperative OR intraoperative OR preoperative OR postoperative OR “operative time” OR “anesthesia recovery period” OR surg* OR “operative room nursing” OR “postanesthesia nursing” |
| APA PsycArticles | robot* AND child* OR pediatric* OR paediatric* OR adolescen* AND pain* OR fear* OR stress* OR anxiety AND perioperative OR intraoperative OR preoperative OR postoperative OR “operative time” OR “anesthesia recovery period” OR surg* OR “operative room nursing” OR “postanesthesia nursing” |
| Web of Science | robot* AND child* OR pediatric* OR paediatric* OR adolescen* AND pain* OR fear* OR stress* OR anxiety AND perioperative OR intraoperative OR preoperative OR postoperative OR “operative time” OR “anesthesia recovery period” OR surg* OR “operative room nursing” OR “postanesthesia nursing” |
| **Other sources** | **Search terms** |
| Mednar | robot* AND (child* OR pediatric* OR paediatric* OR adolescen*) AND (pain* OR fear* OR stress* OR anxiety) AND (perioperative OR intraoperative OR preoperative OR postoperative OR “operative time” OR “anesthesia recovery period” OR surg* OR “operative room nursing” OR “postanesthesia nursing”) |
| Google Scholar | (perioperative OR intraoperative OR preoperative OR postoperative OR “operative time” OR “anesthesia recovery period” OR surg* OR “operative room nursing” OR “postanesthesia nursing”) (robot OR robotics) (child OR pediatric OR adolescent) (pain OR fear OR stress OR anxiety) |

Table S1. Search terms used across databases and other sources.
